# Professional leadership of Chinese university teachers: an empirical exploration of multiple related factors and self-efficacy's role

**DOI:** 10.3389/fpsyg.2025.1677043

**Published:** 2025-10-08

**Authors:** Weiwei Yin, Guowei Zhang

**Affiliations:** ^1^School of Education, Anyang Normal University, Anyang, Henan, China; ^2^School of Mathematics and Statistics, Anyang Normal University, Anyang, Henan, China

**Keywords:** university teachers, personal qualities, school cultural characteristics, self-efficacy, professional leadership

## Abstract

**Introduction:**

Higher education, as the core of talent development and knowledge innovation, relies greatly on the comprehensive capabilities of university teachers, among which professional leadership is vital for the connotative development of universities. Although scholars have researched influencing factors such as individual traits and organizational environment, there are critical gaps in existing studies. This study, set in Chinese universities and based on the Social Cognitive Theory, aims to empirically explore the relationships between Chinese university teachers' personal qualities, school cultural characteristics, and professional leadership.

**Methods:**

Through an exhaustive literature review, seven hypotheses were proposed. A quantitative approach was adopted, data were collected via questionnaires, a structural equation model was established, and data were analyzed using SPSS and Amos.

**Results:**

The results indicate that personal qualities, school cultural characteristics, and self-efficacy are significantly related to professional leadership, with self-efficacy playing a mediating role. A “dual antecedents-single mediator-single outcome” influence path was identified: the two antecedents directly affect professional leadership, and self-efficacy serves as the core bridge.

**Discussion:**

These three factors are crucial for cultivating leadership competencies and institutional advancement. The finding emphasizes the irreplaceability of self-efficacy, breaks the limitation of traditional studies that only focus on the direct impact of personal qualities and school cultural characteristics, and provides key insights into transforming potential conditions into actual leadership behaviors.

## 1 Introduction

Higher education stands as the core domain for talent development and knowledge innovation, and its quality improvement heavily relies on the comprehensive capabilities of university teachers. Beyond the traditional competencies in teaching and research, teachers' professional leadership—such as the roles of instructional guidance, team collaboration, and demonstration and coordination in academic services—has become a key factor in advancing the connotative development of universities (Zhao et al., 2020; Wei and Cheng, 2022; Schott et al., 2020). In recent years, scholars at home and abroad have explored the influencing factors of teachers' professional leadership, identifying individual traits and organizational environment as two core dimensions (Struyve et al., 2018; Liljenberg, 2016; Stout et al., 2017). Nevertheless, existing research still has three critical gaps that urgently need to be addressed.

First, in terms of the systematic nature of variable relationships, most studies only analyze the direct paths from personal qualities to professional leadership or from school cultural characteristics to teachers' professional leadership in isolation, failing to integrate these two dimensions into a unified analytical framework. For example, Zhang et al. (2021) emphasizes the supportive role of teachers' personal competence and communication skills in leadership behaviors, while Kara (2022) focuses on school culture promotes teachers engagement. Neither study, however, addresses the question of whether the combined effect of personal qualities and school cultural characteristics exhibits additive associations with professional leadership, resulting in a fragmented understanding of the underlying influence mechanism.

Second, regarding the exploration of mediating mechanisms, existing research lacks attention to the psychological transmission pathway (Schott et al., 2020). Social Cognitive Theory (Bandura, 1986) posits that individual behavior is not only directly shaped by the external environment and personal qualities but also requires transformation through the core psychological variable of self-efficacy. Specifically, an individual's beliefs about their own capabilities shape the relationship between antecedent variables and behavioral outcomes. While existing studies have supported that teachers' self-efficacy is significantly associated with teaching performance and career development (Liu, 2014; Zhao, 2023), no research has explicitly identified its mediating role in the pathway of “personal qualities/school culture → professional leadership”. This leaves the process of how antecedent variables translate into professional leadership unaccounted for.

Third, in terms of local adaptability, most existing findings are based on the context of universities in Europe and the United States, with insufficient relevance to Chinese universities (York-Barr and Duke, 2004; Wenner and Campbell, 2017; Schott et al., 2020). Chinese universities possess the dual attributes of academic nature and public welfare; teachers face the triple pressures of “teaching–research–service” (Liu et al., 2025); and the school culture is characterized by the coexistence of collective collaboration and hierarchical management (Duan, 2020)—all of which are not entirely the same as the context of universities abroad. Therefore, directly adopting foreign research findings could lead to a lack of alignment with local circumstances. There is an urgent need to systematically explore the influence mechanism and transmission pathway of professional leadership among Chinese university teachers, grounded in the Chinese context.

Based on the aforementioned research gaps, this study takes the teachers of Chinese universities as its research subjects. Centering on the core question of how personal qualities and school cultural characteristics are associated with professional leadership, and whether self-efficacy plays a mediating role therein, it constructs a theoretical model of “antecedent variables–mediating variable–outcome variable”, aiming to address the following three specific research questions:

1. Do the personal qualities (including personal charm, personal competence, and communication skills) and school cultural characteristics (including democracy and respect, trust and collaboration, and encouragement for development) of university teachers each show a positive association with their professional leadership?

2. Do the personal qualities and school cultural characteristics of university teachers each show a positive association with their self-efficacy?

3. Does self-efficacy play a mediating role in both the “personal qualities → professional leadership” pathway and the “school cultural characteristics → professional leadership” pathway?

To achieve the research objectives, the specific connotations of the four important variables in this paper are presented below.

### 1.1 The personal quality of university teachers

The personal qualities of university teachers, including personal charm, personal ability, and communication skills, are of utmost importance in teaching and research (Frost and Harris, 2003; Zhang, 2018; Yan et al., 2022). Personal charm–such as noble virtues, refined manners, high moral standards, a confident and optimistic attitude, and an innovative academic spirit—is essential for teachers. Personal ability, comprising solid disciplinary knowledge and diverse teaching skills, is indispensable. It enables effective knowledge transfer, stimulates students' interest, and fosters their independent thinking. Good communication skills involve efficient interaction and collaboration with various parties and require active listening, clear articulation, and strong organizational abilities. These personal qualities are key to teaching quality and are linked to both teachers' professional development and students' growth (Zhao, 2023; Sun, 2021).

### 1.2 School cultural characteristics

School culture, shaped by interactions among teachers, students, administrators, and campus activities, exhibits distinct characteristics (Barni et al., 2019; Zhang, 2022). In school cultures characterized by democracy and respect, members typically express viewpoints freely while valuing others' perspectives. This pattern of interaction is associated with open teacher-student communication, greater acceptance of diversity, and active engagement in academic exploration. School cultures centered on trust and cooperation are marked by mutual reliance among stakeholders, who collaborate toward shared objectives in teaching, learning, and institutional management. Such collaborative dynamics are often linked to effective coordination across campus functions. In school cultures emphasizing encouragement and development, supportive mechanisms are established to facilitate growth: these include efforts to help students realize their potential and develop critical thinking, as well as initiatives that align with teachers' use of professional skills to support student progress (Zhang, 2022). Such characteristics of school culture are closely linked to the formation of a positive campus environment.

### 1.3 Self-efficacy of university teachers

For university teachers, self-efficacy refers to their beliefs in performing teaching tasks (Liu, 2014). It encompasses confidence in task execution and expectations toward goal achievement. This cognitive-emotional state is shaped by factors such as education background, professional experience, skill proficiency, personality traits, work environment, and feedback, with observable connections to teacher performance, career development, and student learning outcomes. Its characteristics can be specified as follows: it is a form of belief (reflecting subjective perceptions of one's ability to complete tasks); it is self-referential (involving self-evaluation independent of others' judgments); it includes emotional experiences (such as confidence levels in different situational contexts); and it derives from diverse sources of influence (Barni et al., 2019). In practical terms, teacher self-efficacy shows close links to teaching quality and is intertwined with the educational process involving both teachers and students (Liu and Siwatu, 2022).

### 1.4 University teachers' professional leadership

Teachers' professional leadership shows close connections with students, teachers, and schools. For students, it links to knowledge acquisition, skill development, ability enhancement, teacher-student relationships, and non-intellectual growth (Zhao and Ma, 2016). For teachers, it is associated with their own and colleagues' professional development. For schools, it connects to teacher team building, teaching quality, and school visibility (Wei and Cheng, 2022). As a management style, professional leadership is characterized by an emphasis on professional traits, respect for teacher autonomy, promotion of personalized teaching, and cultivation of diverse professional teams (Frost and Harris, 2003). As professionals, teachers leverage their professional strengths such as disciplinary knowledge and non-professional strengths such as personality traits, alongside personal qualities and self-efficacy, to interact with others within a specific school culture. It is a non-administrative, non-mandatory form of leadership rooted in teachers' overall capabilities. Additionally, it is a dynamic form of leadership that evolves alongside teachers' professional advancement (Zhao et al., 2020).

The core purpose of this study is to examine three research questions through empirical methods, clarify the relationships among personal qualities, school cultural characteristics, self-efficacy, and professional leadership, reveal the mediating mechanism of self-efficacy, and construct an influence mechanism model of professional leadership for Chinese university teachers, so as to fill the gaps in systematicity, mediation, and localization of existing research. Specifically, the goal will be achieved by proposing seven specific hypotheses based on Social Cognitive Theory, collecting data via questionnaire surveys and supporting hypotheses with SPSS and Amos, and explaining the formation path of professional leadership based on the results. The study's significance lies in both theoretical and practical aspects: theoretically, it can improve the system of influencing factors of teachers professional leadership, reveal the mediating value of self-efficacy, and fill the gap in localized research; practically, it can provide directions for individual development, offer a basis for university management reform, and serve as a reference for educational administrative departments to design differentiated teachers' leadership development programs for different types of universities and promote the overall improvement of higher education quality.

## 2 Hypotheses

### 2.1 Hypothesis 1

Personal charm and ability are significantly associated with teachers' self-efficacy (Baraczuk, 2021; Furnham and Cheng, 2023). Teachers' demonstration of ethical integrity such as consistent adherence to professional ethics and respectful interpersonal conduct in interactions with students is linked to gaining students' trust, while a confident and optimistic demeanor in teaching scenarios shows connections with positive classroom dynamics (Miao, 2022). Positive student feedback, as noted by Zhao (2023) and Sun (2021), relates to teachers' sense of competence in guiding students, which in turn shows a connection with self-efficacy. Solid subject knowledge is associated with accurate delivery of course content, and diverse instructional skills such as interactive teaching methods relate to sustained student engagement, both of which show links to self-efficacy (Zhao, 2023) . As Barni et al. (2019) highlighted, good communication skills show a consistent connection with self-efficacy: successfully managing communication tasks such as resolving student doubts or coordinating with colleagues relates to greater confidence in one's capabilities, which in turn correlates with self-efficacy. Based on the above, we propose

**Hypothesis 1**: Personal qualities are positively correlated with self-efficacy for university teachers.

### 2.2 Hypothesis 2

School cultural characteristics show significant associations with university teachers' self-efficacy (Karacop and Inaltekin, 2022). Specifically, school cultures emphasizing academic innovation such as institutional support for research exploration and knowledge renewal relate to teachers' perceptions of their academic contribution, which in turn shows connections with self-efficacy (Li et al., 2025). School cultures characterized by trust and collaborative norms such as regular peer support mechanisms for addressing teaching challenges show consistent links to teachers' self-efficacy (Jacobs et al., 2014; Kara, 2022); in such contexts, teachers' experiences of receiving support during difficult tasks correlate with their sense of capability (Rouse, 2021). In school environments with encouraging structures such as resource provision for teaching experimentation and constructive feedback systems, teachers' effective use of available resources to engage students shows associations with greater confidence in their teaching practices, which correlates with self-efficacy (Stout et al., 2017; Rouse, 2021). Based on these observed associations in existing literature, we propose

**Hypothesis 2:** School cultural characteristics are positively correlated with self-efficacy for university teachers.

### 2.3 Hypothesis 3

Teachers' self-efficacy shows significant associations with their professional leadership (Dwyer, 2019; Zhang et al., 2021). Specifically, teachers' beliefs in their capability to perform professional tasks (i.e., self-efficacy) relate to a stronger sense of professional belonging and role clarity, which in turn show connections with their teaching beliefs and behavioral patterns (Zhao and Ma, 2016). Such beliefs also show links to proactive adjustment of teaching practices (e.g., adapting methods to student needs), which correlates with the manifestation of professional leadership (Zhao, 2023). Additionally, teachers' confidence in their professional capabilities (a key aspect of self-efficacy) relates to sustained engagement in knowledge updating, the development of critical judgment in teaching decisions, and the application of innovative strategies (Zhang et al., 2021)—all of which show associations with the demonstration of professional leadership (Zhao, 2023). Based on these observed correlations in existing literature, we propose

**Hypothesis 3:** Self-efficacy is positively correlated with professional leadership for university teachers.

### 2.4 Hypothesis 4

Teachers' personal qualities and their professional leadership capabilities show interactive associations (Frost and Harris, 2003; Zhang et al., 2021; Ying and Ho, 2015). Personal qualities are linked to the foundation of educational quality, while professional leadership connects to educational advancement, with observable mutual correlations between the two (Wei and Cheng, 2022; Kim and Lee, 2020). For university teachers, individual professional competence such as mastery of disciplinary knowledge and teaching methodologies shows consistent links to professional leadership (Zhang et al., 2021). Specific proficiencies in teaching such as designing structured courses and research such as conducting discipline-specific inquiries correlate with the manifestation of leadership capabilities, with such associations extending to outcomes related to student development and academic discipline progress (Struyve et al., 2018; Kim and Lee, 2020; Farhan, 2022). Additionally, personality traits such as adaptability in dynamic teaching contexts and interpersonal skills such as facilitating collaborative learning among students show connections to professional leadership (Fischer, 2020). Based on these observed correlations, we propose

**Hypothesis 4:** Personal qualities exhibit a positive correlation with professional leadership for university teachers.

### 2.5 Hypothesis 5

School environments characterized by specific cultural characteristics-including democratic norms such as inclusive decision-making processes involving teachers in curriculum design, mutual trust such as reliance among colleagues in task coordination, collaborative practices such as regular peer discussion forums, and shared resource access such as shared teaching materials and research databases—show associations with teacher leadership (Hart, 1994; Karacop and Inaltekin, 2022; Kara, 2022). A democratic and harmonious school climate, manifested in open communication channels between teachers and administrators for instance, correlates with teachers' professional leadership capabilities (Wei and Cheng, 2022; Kara, 2022). Respectful interaction norms, such as valuing teachers' input in educational planning, link to teachers' sense of responsibility, while trust-based dynamics, such as autonomy in implementing teaching strategies, correlate with teachers' engagement in leadership roles, with observable mutual connections between these elements (Karacop and Inaltekin, 2022; Chukwry, 2018). Within collaborative frameworks, such as interdisciplinary teaching teams, teachers' participation in professional discourse, refinement of teaching and research skills such as co-developing course modules, and advancement in academic recognition show links to their leadership performance, with these associations mutually reinforcing each other in observable patterns (Duan, 2020; Zhang and Henderson, 2018). Based on these observed correlations in existing literature, we propose

**Hypothesis 5:** School cultural characteristics exhibit a positive correlation with professional leadership for university teachers.

### 2.6 Hypothesis 6 and Hypothesis 7

Drawing upon Bandura's social cognitive theory (Bandura, 1986; Bozack, 2011), self-efficacy functions as a mediating variable that connects an individual's perceived capability to execute specific tasks with behavioral outcomes (Bandura, 1997). Within higher education contexts, self-efficacy shows consistent associations with the relationship between teachers' personal attributes such as adaptability and ethical orientation, professional competencies such as curriculum design skills and disciplinary expertise, and their exercise of professional leadership (Toom, 2018; Zhang et al., 2021). School cultures characterized by supportive elements such as collaborative feedback mechanisms and resource provision for professional growth show associations with self-efficacy as a collective resource, with such cultures linking to enhanced confidence among university teachers (Rouse, 2021; Kara, 2022). Based on these observed associations grounded in social cognitive theory, we propose the following hypotheses:

**Hypothesis 6:** Self-efficacy mediates the relationship between personal qualities and professional leadership for university teachers.
**Hypothesis 7:** Self-efficacy mediates the relationship between school cultural characteristics and professional leadership for university teachers.

## 3 Materials and methods

### 3.1 Participants

A total of 520 questionnaires were collected, among which 403 were valid, resulting in a validity rate of 77.5%. The sample was drawn from 26 Chinese universities across different regions, consisting of 180 males and 223 females. By age, 22.58% were under 36, 38.21% were 36–45, and 39.21% were over 45. In terms of education, 23.33% held a doctorate or post-doc experience, 67.25% had a master's degree, and 9.42% had other backgrounds. Regarding professional titles, 12.9% were professors, 33.5% were associate professors, 42.43% were lecturers, and 11.17% were teaching assistants. For teaching experience, 7.94% had over 29 years, 44.91% had 16–29 years, 35.98% had 6–15 years, and 11.17% had less than 6 years. In terms of position, 11.3% were school administrators, 10.67% were academic leaders, and 78.03% were full-time teachers.

### 3.2 Instruments

**Figure 1:**
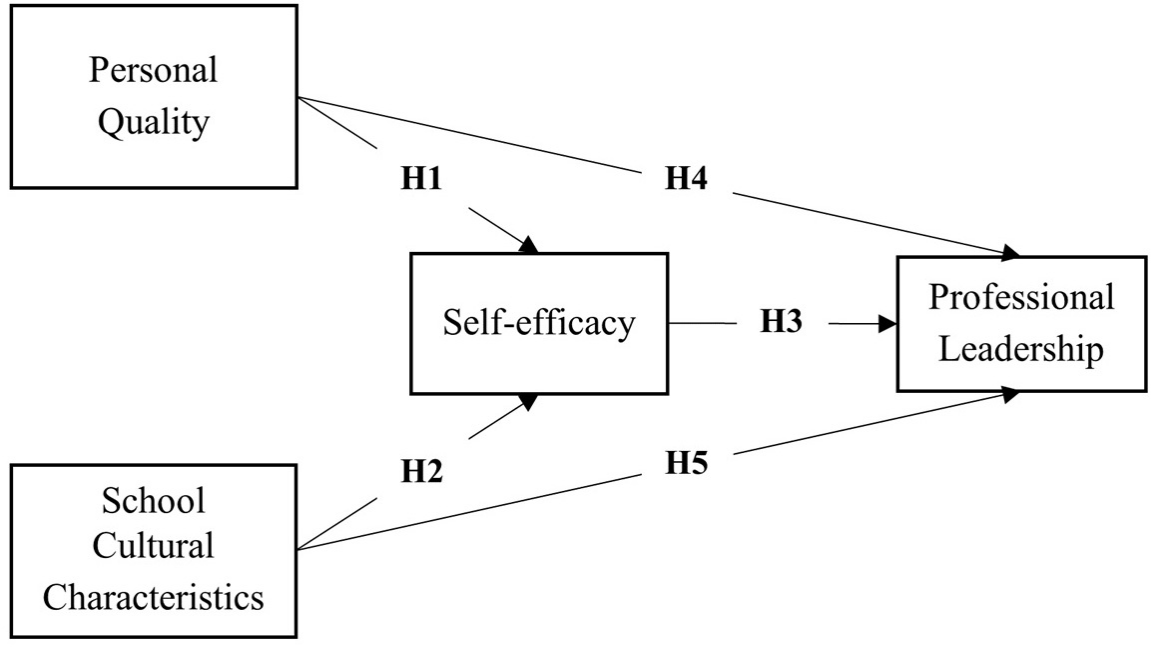
Conceptual framework.

This study used four scales corresponding to the four variables in Figure 1. All scales were adapted from existing relevant scales to be more suitable for Chinese university teachers. For example, the teacher personal qualities and school cultural characteristics scales were adapted from Wang (2011)'s scale; the self-efficacy scale was based on the three dimensions proposed by Bandura (1986) and referenced the self-efficacy scale by Schwarzer et al. (1999); the scale for university teachers' professional leadership was revised by referencing Hu and Gu (2012)'s scale.

During the scale adaptation and development process, a small-sample pre-test was first conducted on the initially developed scales. Exploratory factor analysis was performed on the collected data for each scale, and items with low discrimination or small factor loadings (< 0.35) were deleted, finally forming the scales used in this study.

The teacher personal qualities scale contains 6 items, such as “ I have the awareness and habit of continuously following academic frontier developments and maintaining academic sensitivity.” The school cultural characteristics scale includes six items, such as “In our university, teachers actively participate in group learning and research activities for professional development”. The self-efficacy scale comprises eight items, such as “I think there is no problem that can't be solved by proactive coping, working with others, and asking for help.” The scale for university teachers' professional leadership contains six items, such as “I am willing to lead the team to do a good job in teaching and scientific research and promote the development of my major.” All four scales use a 5-point Likert scale, where 1 = strongly disagree, 2 = disagree, 3 = neutral, 4 = agree, and 5 = strongly agree. Each participant selected the option that best suited them from the five choices following each item.

### 3.3 Research procedure

This study adhered to the Declaration of Helsinki and was approved by the Ethics Committee of the School of Education, Anyang Normal University. Questionnaires were distributed via SoJump (also known as Wenjuanxing) on WeChat. To ensure data accuracy and validity, a convenience sampling method was used at the university department level in this survey. In the introductory section of the questionnaire, we explicitly stressed data confidentiality, clarified that there were no right or wrong answers, and asked participants to respond independently based on their actual situations. All participants submitted their informed consent forms through electronic online means.

### 3.4 Data processing

In this study, SPSS 26 software was utilized to perform descriptive statistical analysis, correlation analysis, and exploratory factor analysis for scale refinement, and finally, the reliability and validity of the data were verified. Subsequently, Amos software was employed for analysis. First, a measurement model analysis was conducted for each latent variable. Next, a structural equation model was constructed, and its fit indices were tested. Finally, based on the modification indices suggestions, a modified structural equation model was obtained. The hypotheses proposed earlier were verified through the analysis results of Amos software.

## 4 Results

To ensure the robustness of the measurement tools and lay a solid foundation for subsequent structural equation modeling, rigorous psychometric testing was conducted using SPSS. First, reliability analysis was performed, and the results showed that all constructs (including Chinese university teachers' personal qualities (PQ), school cultural characteristics (SC), self-efficacy (SE), and professional leadership (PL)) achieved Cronbach's α coefficients greater than 0.8. This exceeds the commonly accepted threshold of 0.7 in social science research, indicating excellent internal consistency within each construct and confirming that the measurement items effectively reflect the same latent variable.

Subsequently, suitability for factor analysis was evaluated through the KMO (Kaiser-Meyer-Olkin) test and Bartlett's sphericity test. The KMO values for all constructs met the standard of being greater than 0.7, while Bartlett's sphericity test yielded significant results (*p* < 0.001), demonstrating that the data had sufficient common variance and were suitable for factor analysis. This provided a reliable basis for further exploring the underlying structure of the measured variables.

The measurement model assessment further confirmed the validity of the scales. Factor loadings of all items on their respective constructs were greater than 0.6, with most exceeding 0.7, and all reached statistical significance at *p* < 0.001. This indicated a strong association between the observed variables and their corresponding latent variables, ensuring that each item effectively measured the intended construct. Additionally, composite reliability (CR) for all constructs exceeded 0.7, which is the recommended threshold for ensuring internal consistency, while the average variance extracted (AVE) was greater than 0.5, meeting the criterion for convergent validity. These results collectively confirmed that the measurement model had good convergent validity, as all constructs sufficiently captured the variance of their respective measurement items (see Table 1).

**Table 1:** The factor loading, CR and AVE.

**Construct**	**Items**	**Standard factor Loading (*p* < 0.001)**	**AVE**	**C.R**.	**Cronbach's α**
PQ	P1	0.757	0.528	0.870	0.871
		P2				0.728			
		P3				0.735			
		P4				0.709			
		P5				0.702			
		P6				0.728			
SC	SC1	0.736	0.551	0.881	0.878
		SC2				0.737			
		SC3				0.745			
		SC4				0.737			
		SC5				0.764			
		SC6				0.736			
SE	SE1	0.755	0.623	0.929	0.929
		SE2				0.737			
		SE3				0.801			
		SE4				0.779			
		SE5				0.708			
		SE6				0.876			
		SE7				0.867			
		SE8				0.777			
PL	L1	0.759	0.524	0.868	0.870
		L2				0.691			
		L3				0.757			
		L4				0.747			
		L5				0.721			
		L6				0.665			

Discriminant validity, which ensures that each construct is distinct from other constructs, was also rigorously verified. For all constructs, the square roots of their AVE values were greater than the maximum correlation coefficients with any other variables in the model. This indicates that each construct shares more variance with its own measurement items than with items from other constructs, further confirming the uniqueness and independence of each latent variable (see Table 2).

**Table 2:** The factor loading, CR and AVE.

	**PQ**	**SC**	**SE**	**PL**
PQ	**0.726**			
SC	0.305	**0.742**		
SE	0.503	0.385	**0.789**	
PL	0.686	0.491	0.640	**0.724**
AVE	0.528	0.551	0.623	0.524

Remark: The bold numbers on the diagonal are the square root of AVEs.

Taken together, the comprehensive psychometric testing–encompassing reliability analysis, suitability for factor analysis, and assessments of convergent and discriminant validity—demonstrated that the measurement scales used in this study are both reliable and valid. These rigorous validation results provide strong support for the subsequent construction and analysis of the structural equation model, ensuring that the model can accurately capture the relationships between Chinese university teachers' PQ, SC, SE, and PL.

**Figure 2:**
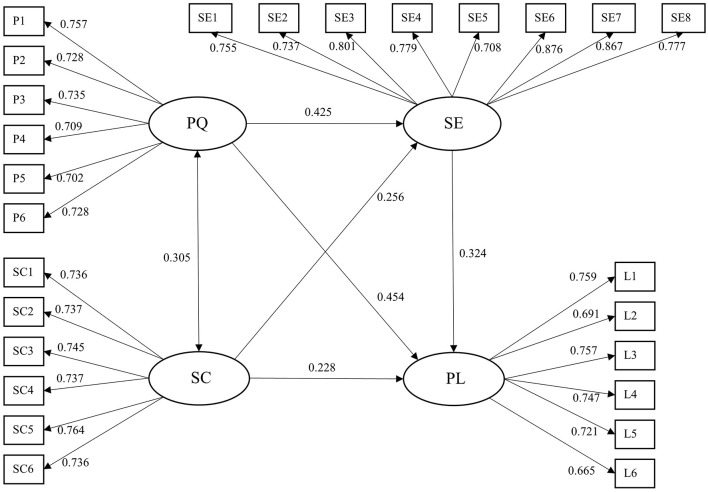
The modified structural equation model.

Based on the modification indices from the Amos analysis, partial error terms were correlated to improve the model fit indices, leading to a modified structural equation model (see Figure 2). The fit indices of the SEM employed in this study are as follows: CMIN/DF = 1.715; GFI = 0.915; AGFI = 0.897; RMSEA = 0.042; SRMR = 0.0477; CFI = 0.965; TLI = 0.961; NFI = 0.921; PNFI = 0.819; PCFI = 0.858. These indices, calculated through the data analysis via SPSS and Amos as part of our quantitative methodological approach, serve as critical indicators to evaluate how well the theoretical model (depicted in Figure 2) aligns with the collected data.

Compared with the commonly accepted cut-off values in academic research, where CMIN/DF is recommended to be less than 3 (ideally less than 2), GFI and AGFI should generally exceed 0.90 (with values above 0.85 often considered acceptable in some contexts), RMSEA and SRMR are expected to be below 0.08 (with values below 0.05 indicating excellent fit), and CFI, TLI, NFI should be greater than 0.90 (Byrne, 2016), all the fit indices in this study meet or exceed these thresholds. Additionally, the PNFI of 0.819 and PCFI of 0.858, which account for model parsimony, also meet the acceptable standards in structural equation modeling.

Such favorable fit indices not only indicate that the model represents the variable relationships–including those between Chinese university teachers' PQ, SC, SE, and PL, as well as the mediating role of self-efficacy–extremely well but also validate the theoretical framework underlying our seven hypotheses. This robust model fit provides solid empirical support for the conclusions drawn from the study, reinforcing the significance of the identified relationships in understanding and cultivating university teachers' professional leadership. For a detailed breakdown of the latent variable relationships, path coefficients, hypothesis testing results, and mediating effect analysis that further corroborate this model fit, refer to Tables 3 and 4.

**Table 3:** The regression weights between constructs.

**Hypothesis**	**Relation**	**Coefficient β**	**S.E**.	**C.R**.	**p**	**Test results**
H1	PQ → SE	0.425	0.072	7.330	***	Support
H2	SC → SE	0.256	0.070	4.817	***	Support
H3	SE → PL	0.324	0.049	6.133	***	Support
H4	PQ → PL	0.454	0.066	7.939	***	Support
H5	SC → PL	0.228	0.058	4.844	***	Support

Remark: ***indicates a significant correlation at the 0.001 level.

**Table 4:** The path coefficient of the mediation effect (standardised).

**Hypothesis**	**Mediation path**	**Effect**	**Effect value**	**Indirect effect**		**Mediation effect**
				**Lower Bounds**	**Upper Bounds**	
H6	PQ → SE → PL	Total Effect	0.591	0.092	0.258	Support
		Direct effect	0.454			
		Indirect effect	0.138			
H7	SC → SE → PL	Total effect	0.311	0.049	0.200	Support
		Direct effect	0.228			
		Indirect effect	0.083			

Table 3 presents a comprehensive overview of the latent variable relationships within the research model, along with detailed path coefficient estimates and critical ratio (C.R.) values. Notably, all path coefficients exhibit statistical significance at the stringent level of *p* < 0.001, which strongly validates the robustness of the associations between the variables under investigation. These variables, including Chinese university teachers' PQ, SC, SE, and PL, along with their interwoven path relationships, constitute the core framework of the theoretical model depicted in Figure 2. This model was developed based on the seven hypotheses derived from our comprehensive literature review and tested through the quantitative methodological approach utilizing SPSS and Amos.

Furthermore, Table 3 explicitly supports that Hypotheses H1 to H5, which were put forward to explore the direct relationships between PQ, SC, and PL, as well as their connections with SE, are all supported by the empirical analysis. This aligns with our overall research findings that these factors are significantly related to professional leadership.

In addition to examining direct effects, the mediating role of self-efficacy was a key focus of this study. To rigorously test this, we employed the Bootstrap method with a 95% confidence interval in Amos, which is a widely recognized approach in quantitative research for assessing mediating effects. The results yielded bias-corrected confidence intervals whose upper and lower bounds did not include zero, a critical indicator supporting the existence of a significant mediating effect. Specifically, this analysis supported the mediation effects proposed in Hypotheses H6 and H7, which pertain to how self-efficacy transmits the influence of PQ and SC to PL. For a detailed breakdown of these mediating effect results, including specific values and statistical indicators, refer to Table 4. These findings collectively underscore the intricate interplay between the variables, reinforcing the conclusion that self-efficacy acts as a crucial mediating mechanism in the relationships contributing to university teachers' professional leadership.

## 5 Discussion

### 5.1 Critical analysis of research results

This study supported all seven hypotheses using SEM, clarifying the relationship mechanism among PQ, SC, SE, and PL. The rationality and limitations of its results require critical examination from three perspectives: the logic of variable relationships, model fit quality, and the boundaries of result interpretation.

#### 5.1.1 Internal logic and rationality of variable relationships

From the perspective of core results, the dual mediating paths of “PQ → SE → PL” and “SC → SE → PL” align with the “trait-environment-psychology-behavior” transmission logic of Social Cognitive Theory (Bozack, 2011; Bandura, 1986), demonstrating significant theoretical consistency.

The positive association between personal qualities and self-efficacy (Hypothesis 1 supported) essentially reflects the accumulative effect of ability perception (Balsdon et al., 2020). Teachers personal charm (e.g., noble professional ethics, innovative academic spirit) and personal competence (e.g., solid disciplinary knowledge, diverse teaching skills) strengthen their belief that they are competent in teaching and leadership task through external signals such as student feedback and colleague recognition (Zhao, 2023; Sun, 2021; Zhang et al., 2021). Meanwhile, communication skills help teachers coordinate teams efficiently and resolve conflicts, further boosting their confidence in professional activities (Baraczuk, 2021)—this is consistent with the theoretical expectation that traits shape self-efficacy through experience accumulation (Waddington, 2023).

The positive association between school cultural characteristics and self-efficacy (Hypothesis 2 supported) embodies the empowering effect of environmental support (Rouse, 2021). A culture of democracy and respect allows teachers to express academic views freely, reducing self-doubt about abilities caused by rejected opinions (Peterson and Jensen, 2025); a culture of trust and collaboration lowers task difficulty through team mutual assistance, enabling teachers to perceive that their abilities can be further amplified through collaboration (Luo et al., 2024); a culture of encouragement for development (e.g., resource support, error-tolerance mechanisms) directly reduces the cost of teachers trial-and-error, making them more willing to attempt leadership behaviors (e.g., leading curriculum reform) (Stout et al., 2017). All these are consistent with the view of (Zhang, 2022) and (Luo et al., 2024) that school culture enhances teachers' confidence by reducing psychological pressure.

The full mediating role of self-efficacy in relation to professional leadership (Hypotheses 6 and 7 supported) reveals the necessity of psychological transformation: if personal qualities and school culture fail to be converted into teachers internal confidence, it will be difficult to form stable professional leadership behaviors (Surucu et al., 2022; Round et al., 2024). For example, a teacher with strong communication skills may be unable to translate such skills into practical leadership behaviors if they lack the confidence to promote team collaboration due to an oppressive school culture (e.g., strict hierarchical management) (Wang and Huang, 2025); conversely, even in an open school culture, a teacher with weak academic charm will struggle to build leadership influence through improved self-efficacy (Jacobs et al., 2014). This result indicates that self-efficacy serves as a key bridge connecting potential conditions (personal qualities and culture) and actual behaviors (professional leadership). Even if the two types of antecedent variables exist objectively, without the transformation by self-efficacy, their impact on professional leadership will struggle to be effectively and efficiently realized due to insufficient transmission. Specifically, they may only lead to scattered and unstable leadership behaviors, failing to translate into sustained and systematic manifestations of professional leadership.

#### 5.1.2 Model fit quality and result reliability

In terms of model fit indices (see Section 4), the mediating model constructed in this study demonstrates an overall good fit, indicating that the results are statistically reliable (Byrne, 2016; Kline, 2005). However, when examining the magnitude of path coefficients, there are still details that require attention.

The path coefficient from personal qualities to self-efficacy (Hypothesis 1) is higher than that from school cultural characteristics to self-efficacy (Hypothesis 2). This may reflect the characteristic of Chinese university teachers that their self-efficacy is more dependent on individual traits. Currently, some universities still adopt an evaluation orientation that emphasizes individual performance over team collaboration, leading teachers to tend to build confidence through improving their own abilities rather than relying on environmental support (Zhang et al., 2021; Ying and Ho, 2015). While this phenomenon aligns with the actual situation of current university management (Wei and Cheng, 2022), it also indicates that the empowering role of the environment in enhancing self-efficacy has not been fully exerted, which needs further optimization in practice.

The path coefficient of self-efficacy on professional leadership (Hypothesis 3) falls between the direct path coefficients of personal qualities and school cultural characteristics on professional leadership (Hypotheses 4 and 5), which further supports the core value of the mediating role. This implies that relying solely on the direct impact of personal qualities or school cultural characteristics on professional leadership can have some influence, but the effect is relatively limited (Zhang et al., 2021; Rouse, 2021). The intervention of self-efficacy optimizes this influencing process. It enables teachers to, based on a positive perception of their own abilities, more efficiently translate the potential advantages brought by personal qualities and school culture into practical professional leadership actions (Wang and Huang, 2025; Round et al., 2024). For example, teachers take the initiative to play a leading role in scenarios such as curriculum design, team collaboration, and academic promotion, forming a stable and continuous output of professional leadership (Zhang and Henderson, 2018). The moderate path coefficient of self-efficacy precisely highlights its crucial boosting value in integrating individual—environment resources and promoting the formation of professional leadership. It also supports its core position in the research model from a quantitative perspective. That is, it does not simply strengthen the impact of antecedent variables on professional leadership, but rather reshapes the impact path, making the entire mechanism more reasonable and efficient.

#### 5.1.3 Boundaries of result interpretation and potential biases

Despite the rationality of the results, their interpretive boundaries must be clarified to avoid overgeneralization. First, there are limitations in sample representativeness. The study obtained 403 valid questionnaires from 26 universities, but the regional distribution was not explicitly balanced, and the proportion of different types of universities (e.g., research-oriented, application-oriented) was not specified. Additionally, the teachers educational background (23.33% with doctorate/post-doc, 67.25% with master's) and professional title structure may not fully match the national situation of Chinese universities (MyCOS-Research, 2025), potentially restricting the generalizability of results. Second, measurement biases exist (Zhou and Long, 2004; Podsakoff et al., 2003). The measurement of professional leadership only covered instructional guidance, team collaboration, and academic services, excluding dimensions like participation in discipline construction decision-making—this may underestimate the impact of some antecedent variables. Meanwhile, the self-report method (Rosenman et al., 2011) is prone to social desirability bias such as university teachers overestimating their abilities, requiring verification with multi-source data in future studies.

### 5.2 Comparison with other related research findings

The results of this study have a three-fold relationship of echo–supplementation–expansion with the existing literature, further highlighting the academic value of the study.

#### 5.2.1 Echo with direct relationship research

In the direct paths of “PQ → PL” (Hypothesis 4 is supported) and “SC → PL” (Hypothesis 5 is supported), the results of this study are highly consistent with the previous literature.

Regarding the influence of personal qualities, Zhang et al. (2021) found through research on Chinese universities that teachers' communication ability and academic charm can significantly enhance their influence in the team. Duan (2020) also pointed out that personal qualities such as responsibility and open mindedness of Chinese university teachers are positively correlated with leadership behavior. This is completely in line with the conclusion of this study that personal charm, personal ability, and communication ability positively predict professional leadership, indicating that the supporting role of personal qualities in professional leadership has cross-situational consistency.

Regarding the influence of school culture, Zhou and Guo (2014)'s research on Chinese universities shows that a culture of trust and collaboration can promote knowledge sharing among teachers, thereby enhancing the overall leadership effectiveness. Li et al. (2025) proposed for Chinese universities that a supportive academic culture can stimulate teachers' initiative in leadership. This is consistent with the result of this study that a culture of democracy and respect, trust and collaboration, and encouragement of development positively predicts professional leadership, supporting the universality of the influence of school culture (Shen and Tian, 2012).

#### 5.2.2 Supplementation to mediation mechanism research

In terms of the mediating role of self-efficacy, this study fills the gap in existing research and forms an important supplement. Existing research mostly focuses on the mediating role of self-efficacy in teaching behavior and job burnout (Liu, 2014; Liu et al., 2022), but has not extended to the field of professional leadership. This study for the first time supports the complete mediating role of self-efficacy between PQ/SC and PL, supplementing the empirical evidence of the influence of teachers' psychological variables on leadership behavior and echoing Bandura (1986)'s theoretical expectation that self-efficacy is the core mechanism of behavior transformation.

Compared with the research that personal qualities are positively associated with self-efficacy (Baraczuk, 2021; Furnham and Cheng, 2023) and that school culture is positively associated with self-efficacy (Li et al., 2025; Rouse, 2021) , this study further associates self-efficacy with professional leadership, constructing a complete chain of “antecedent–mediator–outcome”, upgrading the variable relationship from unidirectional influence to system mechanism, and deepening the understanding of the formation path of teachers' professional leadership.

#### 5.2.3 Expansion of localization research

In terms of the adaptability of the research context, this study has carried out localized adaptation of the research conclusions based on international contexts.

Studies on foreign universities often emphasize the impact of teacher autonomy on professional leadership (Lin and Gao, 2023; Frost and Harris, 2003). However, through data analysis, this study finds that personal qualities, school culture characteristics, and self-efficacy all have significant impacts on the professional leadership of Chinese university teachers. Specifically, the impact coefficient of personal qualities on professional leadership is 0.454, that of school culture characteristics is 0.228, and that of self-efficacy is 0.324. This indicates that self-efficacy does not play an exclusive leading role among the three factors; instead, it exerts an influence on professional leadership together with personal qualities and school culture characteristics.

The collectivist culture (Tan et al., 2021; Zhang and Han, 2023) in Chinese universities makes teachers pay attention to the alignment between their own abilities and the expectations of the team and the university (Yang, 2016). In this process, self-efficacy, as one of the influencing factors, interacts with personal qualities and school culture characteristics to jointly promote teachers to demonstrate professional leadership in instructional guidance, team collaboration, and academic services. Therefore, this study does not present a scenario where self-efficacy acts as the sole mediator; rather, it reveals the synergistic influence of multiple factors. This finding revises the foreign research conclusions that overemphasize individual autonomy, making them more suitable for the Chinese context.

### 5.3 The connection between theoretical significance and practical implications

The theoretical findings and practical implications of this study are closely linked, presenting a relationship of theory guiding practice and practice feeding back to theory.

First, theoretically, it is supported that self-efficacy is a full mediating variable connecting personal qualities (school culture) and professional leadership. This directly guides the design of psychological empowerment strategies in practice (Meng and Sun, 2019). Teachers can enhance their self-efficacy by accumulating small successes and participating in experience-sharing sessions (Lopez-Garrido, 2025), while universities need to establish feedback mechanisms and integrate incentive elements into their culture. Second, theoretically, an individual-environment interaction model (Kristof-Brown, 2020) is constructed, breaking the limitation of single-dimensional analysis and guiding the collaborative practice of trait improvement and cultural optimization: for universities with high personal qualities but low culture, priority should be given to optimizing the environment to unlock the potential of traits; for universities with low personal qualities but high culture, focus should be placed on strengthening teachers qualities training. Third, theoretically, a localized influence mechanism is verified, which breaks the limitation of directly applying foreign experiences, and guides context-specific targeted measures: In line with Chinese universities collectivist culture (where teachers align personal abilities with team/university expectations), research-oriented universities should focus on fostering teachers academic charm and an academic democratic culture; application-oriented universities, meanwhile, should prioritize enhancing teachers communication skills and a collaborative culture. These strategies avoid mismatches from overemphasizing foreign style individual autonomy, fitting the actual context of Chinese higher education.

The implementation of practical paths can also feed back to the improvement of theories. For example, if it is found in the practice of psychological empowerment that there are differences in the influence mechanisms of self-efficacy among teachers with different teaching ages (Sun and Yin, 2025), the teaching age can be added as a moderating variable to the theoretical model; if it is found in the practice of targeted measures that the intensity of cultural influence differs between private and public universities (Zhang et al., 2021), the contextual boundaries of the theory can be further expanded, and the differences in strategies among universities of different ownership types can be explored to provide a new perspective for the theory.

This two-way feedback between theory and practice can continuously optimize the cultivation system of university teachers professional leadership, contribute to the improvement of higher education talent training quality, and form a positive cycle of theory leading practical exploration and practice promoting theoretical deepening. It promotes the in-depth development of research and practice in this field and provides more solid theoretical support and more operable plans for the reform of university education and teaching.

## 6 Conclusion

Based on the context of Chinese universities and framed by Social Cognitive Theory, this study systematically explored the relationship mechanism among university teachers PQ, SC, SE, and PL through empirical analysis. The core research findings and values can be summarized as follows.

In terms of the variable relationship mechanism, the study identified a complete influence path characterized by “dual antecedents–single mediator–single outcome”. While university teachers personal qualities (including personal charm, personal competence, and communication skills) and school cultural characteristics (including democracy and respect, trust and collaboration, and encouragement for development) have a direct impact on professional leadership, self-efficacy serves as the core bridge through which the two act on professional leadership. More importantly, by strengthening teachers belief in their own abilities via this mediating path, the two factors significantly promote teachers to demonstrate professional leadership in instructional guidance, team collaboration, and academic services, which highlight the key value of the mediating effect in the variable relationship. This finding reveals the irreplaceability of self-efficacy in the formation of teachers professional leadership, breaks the limitation of traditional studies that only focus on the direct impact of external conditions (traits or culture), and provides key insights for understanding how potential conditions are transformed into actual leadership behaviors.

Regarding theoretical contributions, the value of this study is reflected in three aspects. First, it is the first to construct and test an integrated “individual–environment–psychology-behavior” model in the context of Chinese universities, filling the gap of isolated analysis of individual and environmental variables in the research on teachers professional leadership. Second, it empirically confirms the full mediating role of self-efficacy, providing localized empirical support for the application of Social Cognitive Theory in the field of higher education teachers leadership research and enriching the cross-context explanatory power of this theory. Third, targeting the cultural characteristics of collective collaboration and supportive development in Chinese universities, it revises research conclusions based on foreign contexts, promoting the deepening of teachers professional leadership theory toward localized adaptation.

As for practical value, the research conclusions provide actionable hierarchical paths for the cultivation of university teachers professional leadership: For individual teachers, they should simultaneously focus on the proactive improvement of personal qualities (such as academic charm and communication skills) and the positive enhancement of self-efficacy, gradually building confidence by first engaging in trial and error with low-difficulty leadership tasks, then accumulating positive feedback from these experiences. For university administrators, priority should be given to building a supportive cultural ecosystem featuring democracy, trust, and encouragement, and providing environmental support for trait transformation and self-efficacy improvement through institutional design (such as teachers academic decision-making rights and interdisciplinary collaboration platforms). For educational administrative departments, differentiated cultivation programs can be designed for different types of universities (research-oriented, application-oriented) based on the research conclusions to achieve the coordinated improvement of “traits–culture–efficacy–leadership”, ultimately serving the connotative development of higher education quality.

It should be objectively noted that the limitations of this study in terms of samples and methods (e.g., cross-sectional design, self-reported data) leave room for expansion in future research. Subsequent studies can further verify the stability of the model while providing more empirical evidence to support the inference of causal relationships between variables by expanding the sample coverage, adopting cross-lagged designs and mixed research methods, and deeply explore the moderating role of variables such as teaching age and discipline type, so as to provide more comprehensive and in-depth empirical evidence for the research on university teachers professional leadership.

## Data Availability

The original contributions presented in the study are included in the article/supplementary material, further inquiries can be directed to the corresponding author.
